# The long way to diagnosis: attention disorder, alcohol addiction or congenital disorder of glycosylation? A case report

**DOI:** 10.1186/s12888-025-06862-9

**Published:** 2025-04-29

**Authors:** Timo Jendrik Faustmann, Lukas Hensel, Armin Bahic, Yoshinao Wada, Marianne Grüneberg, Thorsten Marquardt, Daniel Kamp, Leonhard Schilbach

**Affiliations:** 1 Department of Psychiatry and Psychotherapy, Medical Faculty, Heinrich Heine University Düsseldorf, Universitätsstraße 1, 40225 Düsseldorf, Germany; 2 Department of General Psychiatry 2, LVR-Klinikum Düsseldorf, Bergische Landstraße 2, 40629 Düsseldorf, Germany; 3 Department of Psychiatry and Psychotherapy, University Hospital, Ludwig Maximilians University Munich, Nußbaumstraße 7, 80336 Munich, Germany; 4 Department of Neurology, Medical Faculty, University Hospital Cologne, University of Cologne, Kerpenerstraße 62, 50937 Cologne, Germany; 5 Department of General Pediatrics, University Children’s Hospital Münster, Albert-Schweitzer-Campus 1, 48149 Münster, Germany; 6 Department of Molecular Medicine, Osaka Medical Center and Research Institute for Maternal and Child Health, 840 Murodo-cho, Izumi, Osaka, 594-1101 Japan

**Keywords:** ADHD, CDT, CDG, Case report, Psychostimulants

## Abstract

Attention deficit hyperactivity disorder (ADHD) is a relatively common disorder in clinical psychiatry. Patients often suffer from symptoms long before the diagnosis due to an overlap with other psychiatric differential diagnosis. Importantly, alcohol addiction and other illicit drug dependence and withdrawal symptoms mimicking ADHD symptoms should be ruled out. Here we present a rare case of a young female patient with symptoms of ADHD and an extremely high carbohydrate-deficient transferrin (CDT) of 19,6% (< 1,3%) indicating the presence of a congenital disorder of glycosylation (CDG). A thorough diagnostic workup excluded alcohol addiction as a cause of the constantly high CDT levels. The CDT test was positive due a transferrin mutation affecting the glycosylation site. Nevertheless, psychiatric symptoms can be due to metabolic disorders which should be considered. Further, substance-use disorders (SUD) are a critical and potentially complicated differential diagnosis concerning diagnostic procedures and treatment in ADHD.

**Supplementary Information:**

The online version contains supplementary material available at 10.1186/s12888-025-06862-9.

## Background

### ADHD

Attention deficit hyperactivity disorder (ADHD) is a neurodevelopmental disorder that begins in childhood and persists into adulthood in up to 15–60% of patients. Depending on the age, approximately 2–5% of the population is affected by ADHD [1–3]. Core symptoms include inattentiveness, impulsivity and hyperactivity. Progressing from childhood to adulthood, hyperactivity usually turns into an inner restlessness in adults [4]. Moreover, patients may present with comorbidities like substance-use disorders (SUD) which are very comment in ADHD and further are potentially misdiagnosed with other conditions e.g. mood disorders or personality disorders [2, 5, 6]. According to German guidelines the diagnosis of ADHD can be made after evaluation of the clinical presentation and standardized psychometric testing [7]. Standardized instrumental diagnostics is not recommended, but commonly routine blood parameters, urine toxicology screening, brain magnetic resonance imaging (MRI) and carbohydrate-deficient transferrin are used to evaluate the somatic differential diagnosis and potential substance abuse and should be taken into account before starting a medication [7]. Further in case of consumption (e.g. alcohol) abstinence during the diagnostic process is important but often not possible [8, 9]. Treatment of ADHD is multimodal including psychostimulants with significant effects on symptom control, psychoeducation, psychotherapy and neurofeedback training [7].

### CDT

Transferrin is an iron-binding glycoprotein transporting iron throughout the human body. Carbohydrate-deficient transferrin (CDT) is mainly known as a long-term marker for alcohol abuse and describes transferrin isoforms which are deficient in their structure and appear after ethanol consumption of about 50–80 g/d for more than 10–15 days. As studied in 2500 individuals, the sensitivity for alcohol consumption was 82% and the specificity was 97%, respectively. The reason for the hypoglycosylation is not clear but could be an acetaldehyde-mediated inhibition of glycosyl transfer [10–12]. CDT is important for assessing possible alcohol dependence and differentiating alcohol abuse from enzyme-inducing medication [13]. Variations of CDT levels have been associated with pregnancy [14], fructose intolerance and galactosemia [15] and chronic obstructive pulmonary disease (COPD) [16]. Further reasons for an increase in CDT could be genetic variants of transferrin, chronic active hepatitis, primary biliary cirrhosis and congenital disorders of glycosylation (CDG) [10].

### CDG

CDG are a heterogeneous group of genetic disorders that include various defects in the processing or synthesis of glycoproteins [17]. Around 160 genetic conditions have been described commonly leading to dysfunctional hypo- or dysglycosylation of lipids and proteins [18, 19]. Symptoms range from mild to severe including the failure to thrive, intellectual disability, developmental delay, facial dysmorphism, muscular hypotonia, ataxia due to cerebellar atrophy, epilepsy, liver disease, endocrinopathies, retinopathy, coagulopathy and hypoglycemia [20, 21] Prevalence in Europe is around 0.1–0.5/100.000 but appears to be underdiagnosed [22]. The diagnostic workup consists of a CDT screening, transferrin isoform analysis with high-performance liquid chromatography (HPLC), protein-linked glycan analysis with mass spectroscopy and genomic sequencing. Yet, biochemical pathogenesis is diverse [20, 23] and to date, mainly four groups are distinguished: disorders of N- linked glycosylation, disorders of O-linked glycosylation, combined N- and O-linked/multiple disorders of glycosylation and lipid and glycosylphosphatidylinositol anchor biosynthesis defects [24].

CDG type I affecting the glycan synthesis and CDG type II affecting glycan processing are the subdivisions of disorders of N-glycosylation [25]. The treatment depends on the exact form of CDG. Basically, three basic treatment concepts have been suggested: substrate (precursor) supplementation, cofactor supplementation and pharmacological chaperons. Further, non-causative and other treatments have been discussed [24]. For example, oral mannose can be used for mannose-6 phosphate isomerase- (MPI-) CDG, galactose for phosphoglucomutase-1- (PGM1-) CDG to increase coagulation factors and decrease serum transaminase levels, liver transplantation in MPI-CDG and coiled-coil domain containing 115- (CCDC115-) CDG, bone marrow transplantation in phosphoglucomutase-3- (PGM3-) CDG and in further variants treatment of specific symptoms like pericarditis, hypothyroidism and hypoglycemia [20, 22].

The here presented case points towards interesting overlaps of ADHD symptoms, SUD and metabolic diseases.

## Case presentation

### Instrumental diagnostics

Written informed consent was obtained from the patient for the publication of this case report. The CARE guidelines for case reports were followed [26]. Routine blood parameters including CDT were performed. Additionally, urine toxicology screens including ethyl glucuronide were conducted. Genetic screening for a mutation of Aldolase B gene and Transferrin gene was performed. Breath alcohol and Clinical Institute Withdrawal Assessment for Alcohol-Revised (CIWA-Ar) score was used to rule out signs of alcohol intoxication and withdrawal [27]. Transferrin isoforms were analyzed using HPLC. Liver MRI, electrocardiogram, internal medicine consultation, abdominal ultrasound and neurological examination were additionally performed.

### Psychometrics

For ADHD testing we used the world health organization (WHO) Adult ADHD Self-Reported Scale (ASRS-V1.1). The self-report screening questionnaire consists of 6 questions (minimum 4 questions with significant intensity required) asking for the severity of inattention, hyperactivity, and impulsivity symptoms listed in Diagnostic and Statistical Manual of Mental Disorders 4th edition (DSM-IV) [28] and ADHS-SB/HASE-SB (ADHS- Selbstbeurteilungsbogen/Homburger ADHS-Skalen für Erwachsene Selbstbeurteilungsbogen) self- disclosure questionnaire consisting of 18 questions (Cut-Off for significant ADHD symptoms = 18 points) [29, 30] followed by a standardized interview in German language - the integrated diagnosis of ADHD in adults revised version (IDA-R) - including a semi-structured interview (9 questions on attention-deficits and 9 questions on hyperactivity, minimum 5 questions with severe symptoms required for attention-deficits and minimum 5 questions with severe symptoms required for hyperactivity) to evaluate if symptoms meet the criteria according to Diagnostic and Statistical Manual of Mental Disorders 5th edition (DSM-5) [31]. Wender-Utah rating scale (German short version, WURS-K, 25 questions, Cut-Off for significant ADHD symptoms during childhood ≥ 30 points) was used to evaluate childhood symptoms of ADHD [32, 33]. Further, written teacher school reports from elementary school (grades 1–4) were screened giving an impression of how the patient behaved in elementary school. Multiple Choice Word Test-B (MWT-B) consists of 37 questions and was used for the intelligence diagnostic (age validated intelligence quotient) [34]. Becks Depression Inventory II self-report questionnaire (BDI-II) comprising 21 questions was used to evaluate symptoms of depression (0–8 points = no depressive symptoms, 9–13 points = minimal depressive symptoms, 14–19 points = mild depressive symptoms, 20–28 points = medium depressive symptoms, > 28 points = severe depressive symptoms) [35, 36]. D2 concentration-endurance test (D2 test, validated by age, concentration can be above average, average or below average) [37] was used to evaluate the concentration. The German version of PSDI (Personality Styles and Disorder Inventory) consists of 140 items evaluating the non- pathological equivalents of 14 personality disorders in DSM IV and International Statistical Classification of Diseases, 10th revision, German Modification, Version 2024 (ICD-10-GM Version 2024) and was used to evaluate signs of personality accentuation (validated by age, t-values between 40 and 60 are average) [38]. Autism Quotient (AQ) consists of 50 items [39] and was used to differentiate ADHD symptoms from possible symptoms of an autism spectrum disorder (Cut-Off for significant symptoms ≥ 32 points). Global assessment of function (GAF) was used to evaluate the general level of psychosocial functioning (Score ranges from 1 (severe impairment in psychosocial functioning) to 100 (high psychosocial functioning)) [40].

### Case

**Fig. 1:**
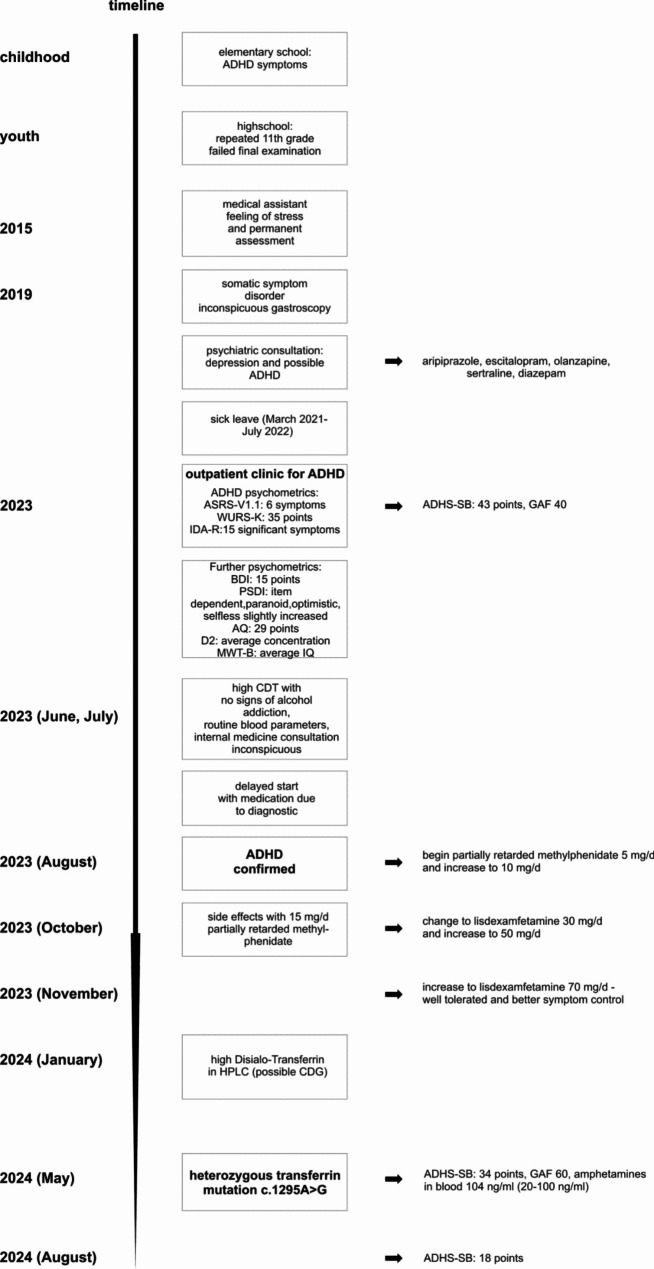
Timeline of symptoms and diagnostic procedure

A 30-year-old Turkish descendent, medical assistant, female patient was transferred by a psychiatrist to our specialized outpatient clinic in 2023 with symptoms of ADHD (difficulties in reading and writing, focusing, concentration, feelings of stress and permanent assessment) for completion of an ADHD diagnostic. She received no medication when she arrived at our department for the first time. The patient reported no other somatic diseases. There was no family history of severe physical or mental illnesses. The patient reported a history of depression and suspicion of ADHD, but without having received a work-up for ADHD. The patient had been on a previous medication of sertraline, escitalopram, aripiprazole, olanzapine and diazepam, but without a psychiatric inpatient treatment. Before she was put on sick leave by her general practitioner because of a somatic symptom disorder in 2021 and 2022 (Fig. 1). At the time of consultation at our clinic a somatic symptom disorder could not be diagnosed. She reported symptoms of attention deficit and difficulties in focusing since her youth. School reports from elementary school (grades 1–4) revealed strong symptoms of ADHD including inattention, easy distraction by other pupils, concentration difficulties, aid and additional time for tasks, problems implementing complex tasks, not targeted, inaccuracy, and difficulties in writing. She failed her final examination in high school and had to repeat the 11th grade. During the time of consultation in our clinic the patient continued working as a medical assistant. During the diagnostic process in our outpatient clinic, she revealed the following scores in Table 1:

Mental status exam revealed a patient oriented to person, place and time with cooperative behavior. The motor activity revealed some psychomotor agitation and some fidgeting with her hands. The attention span was reduced and the concentration was mildly impaired. The thought form was diffuse. The affect was appropriate. The mood was euthymic. The thought content was normal. The patient denied suicidal or homicidal ideations. Neurological examination was unremarkable.

In total the diagnosis of ADHD combined type was made according to the German guidelines for ADHD, the European consensus statement and ICD-10-GM Version 2024 without comorbidities like depression, autism, personality disorders, psychotic disorders or somatic symptom disorders according to ICD-10 [7, 41, 42].

In parallel before starting ADHD-medication routine blood parameters including blood cell count, coagulation, liver enzymes, kidney parameters, electrolytes and thyroid parameters were normal. Monocytes were slightly increased to 10.8% on the 22nd of June 2023, 11.4% on the 28th of July and 10.8% (2.0-9.5) on the 10th of August 2023. Lactate dehydrogenase (LDH) was 288 U/l (< 250) on the 28th of July 2023. Immunofixation was negative. Anti-nuclear antibodies (ANA), anti-mitochondrial antibodies (AMA), anti-soluble liver antigen antibodies (SLA), liver kidney microsome antibodies (LKM), anti-smooth muscle antibodies (ASMA), cytoplasmic anti-neutrophil cytoplasmic antibodies (c-ANCA), perinuclear ANCA (p-ANCA) were all negative. Atypical ANCA (X-ANCA) were 1:32 (1:<10). Serum protein electrophoresis was negative. Beta1-globulin was 4,6% (4,9 − 7,2). Transferrin was 1,65 g/l (1.80–3.82) and ferritin was 47,7% (16–45%) on the 28th of July 2023.

An unusually high CDT of 19.6% (< 1,3%) was found on the 23rd of June 2023 followed by 19,1% on the 27th of June 2023 and 23,2% on the 10th of August 2023. Yet, urine ethyl glucuronide was negative (< 0,5 mg/l) on the 14th of July 2023 and 10th of August 2023, repeatedly indicated absence of alcohol intake. Urine toxicology screening was also negative for opioids, cannabinoids, amphetamines and cocaine on the 27th of June 2023, 28th of July 2023 and 10th of August 2023. Breath alcohol and CIWA-Ar score was negative on the 27th of July and 28th of July. The anamnesis by the husband also was not suggestive of addiction. In conclusion, after repeated consultations with the patient and her husband and consistent laboratory results, alcohol addiction was ruled out. Pregnancy test was negative on the 27th of June 2023.

In parallel, because of the high CDT transferrin isoforms were analyzed using HPLC and revealed high Disialo-Transferrin of (22,8% (< 1,8%)) and low Tetrasialo-Transferrin (70% (> 85%)). Other parameters revealed 0.0% of Monosialo-Transferrin (0.0%), 2,4% of Trisialo-Transferrin (< 6.5%), and 4,8% of Pentasialo-Transferrin (< 15%) respectively, pointing towards evidence for a congenital disorder of glycosylation.

Additionally, genetic testing ruled out hereditary fructose intolerance, a known cause of transferrin hypoglycosylation. Native liver MRI-scan was normal; the patient rejected receiving contrast agent. Abdominal ultrasound and internal medicine consultation were negative for any abdominal and internal medicine abnormalities. Electrocardiogram was normal. An additional MRI scan of the brain was rejected by the patient.

After extensive diagnostic procedures in August 2023, partially retarded methylphenidate was started with 5 mg/day and increased up to 10 mg/day and later 15 mg/day. This was not well tolerated, and the patient reported headache, agitation and nausea so that the medication was changed to lisdexamfetamine in a dosis of 30 mg/day due to mentioned side effects. The dosis was increased to 50 and later on to 70 mg/day by the beginning of 2024. Symptoms decreased and the patient felt better during the day. Drug levels revealed 104 ng/ml amphetamines (20–100 ng/ml) in the blood on the 29th of May 2024 and symptom scores in the ADHS-SB decreased (from 43 in 2023 to 34 in May 2024 and 18 in August 2024). Further, GAF improved from 40 in 2023 (Burdened by psychosocial stress related to work) to 65 in 2024 (Improvement at work, finished exam at work) (Table 1).

Since the patient improved with lisdexamfetamine but still reported symptoms a further diagnostic and therapy regarding CDG was recommended but rejected by the patient. The patient wanted to postpone the decision of further diagnostic into the future if symptoms remain.

In May 2024 transferrin analysis revealed the heterozygous transferrin mutation c.1295 A > G that destroys the glycosylation site at the asparagine 432. The amino acid changes due to the mutation as well as the lack of glycan at this site were confirmed by mass spectrometry. Thus, transferrin in the patient has only one glycosylation site left and the hypoglycosylation is not indicative of CDG disease.

## Discussion

In this case we report a young female patient who initially presented with symptoms of ADHD. The case is unique because of the following reasons:

1. The abnormally high serum CDT was initially considered as a likely indicator for an overlap with a possible alcohol dependence.
2. Stigmatization of psychiatric patients due to false positive biomarkers.
3. These findings led to a delayed start of the ADHD therapy because of the more complex differential diagnosis for rare causes of elevated CDT.
4. Diagnostic/Treatment of ADHD and co-morbid substance abuse.
5. Co-existing ADHD together with CDG (which was suspected in first place) and possible role of glycosylation related to ADHD.
6. Lisdexamfetamine was a good treatment option in this case of ADHD.
7. Complex possible metabolic and genetic findings in psychiatric disorders.

### Reason 1–4: problems of co-existing ADHD and SUD

The complex situation of ADHD and SUD includes the problems of an adequate diagnostic under possible substance abuse. It can be discussed that during the diagnostic procedure in cases of addiction a first screening for ADHD symptoms can be done but complete testing should be performed after withdrawal cause intoxication and withdrawal symptoms can mimic ADHD symptoms [8, 9]. Further, the treatment with a substance like methylphenidate and lisdexamfetamine (psychostimulants) potentially is discussed to be misused in e.g. 5–35% of cases in high school students and college students and abuse and diversion potential can be discussed. Further, additionally effects on the cardiovascular system like mild increase in blood pressure and heart rate need to be discussed and are important if further psychiatric and somatic comorbidities exist [43–46]. These facts have to be taken into account if in parallel a continued intake of another substance due to an abuse exist which further might complicate the therapy [9]. In conclusion, a close screening and monitoring of ADHD medication use is recommended [44]. Around 50% of patients with ADHD have SUD and the role of “self-medicated” ADHD symptoms can be discussed. The SUD therapy can suffer under a not treated ADHD and ongoing substance abuse can limit the ADHD therapy. The use of non-stimulant pharmacotherapy needs to be discussed for cases of ADHD and SUD [9, 47]. Interestingly, psychostimulants are discussed to reduce risky behavior like substance abuse among patients with ADHD and prevent the development of SUD, but physicians often are not sure about the exact treatment of patients presenting with ADHD and substance abuse at the same time [48]. In a meta- analysis comparing follow up studies the use of psychostimulants in childhood was related to a reduction in the risk for drug and alcohol addiction later in life [49]. Further, different administration (e.g. oral, intravenous) revealed that orally intake of methylphenidate constrains abuse [50]. Further, the occurrence of e.g. possible lisdexamfetamine abuse has been discussed but the concerns could not be substantiated in patients [51].

### Reason 5: co-existing ADHD together with CDG (which was suspected in first place) and possible role of glycosylation related to ADHD

To date, only few cases have linked abnormal glycosylation to attention disorders including ADHD. For example, alpha-1,3-glucosyltransferase- (ALG8-) CDG has been found to show intellectual disabilities besides other organ manifestations including musculoskeletal, dermatologic and cardiac symptoms [52]. Further, alterations of glycan composition of glycoproteins were found in patients with ADHD and it was discussed that these alterations are connected to further findings of patients with the diagnosis of CDG and changes in behavior and neurological activity and variants in fucosyltransferase 8 (FUT8) at the same time [53, 54]. Another case with Conserved Oligomeric Golgi (COG-)CDG was reported to include symptoms of ADHD in the clinical presentation [55]. Taken together the exact pathomechanism of symptoms of ADHD occurring together with a possible CDG is not clear. However, the association may be overlooked if standard serum markers such as liver enzymes show normal serum levels or in patients with overlapping substance abuse. Previously, neurodevelopmental disorders such as autism have been described in CDG. Further N-glycosylation abnormalities were described in ADHD [56, 57]. Concerning the here described symptoms of ADHD together with a suspected occurrence of CDG at the same time one mechanism of pathology can be a missing N-linked glycosylation which is important for the membrane localization of the dopamine D5 receptor [58]. This receptor was discussed to be important in ADHD [59]. Further, contradicting glycosylation of the dopamine transporter (DAT) is more efficiently in transporting dopamine and was discussed to be involved in the vulnerability of midbrain dopamine cells in Parkinson’s disease [60]. Since dopamine and DAT are one therapeutical target in ADHD treatment this could be one possible mechanism when considering ADHD and CDG at the same time.

### Reason 6: lisdexamfetamine was a good treatment option in this case of ADHD

In the present case, treatment with methylphenidate was not well tolerated. Lisdexamfetamine was a good alternative option and the patient reported symptom relief with 30 mg and later 70 mg. Lisdexamfetamine is a long-acting amfetamine prodrug and approved by the FDA (US Food and Drug Administration) and EMA (European Medicine Agency) for ADHD in adults inhibiting the dopamine and noradrenaline transporters, inhibiting the transmitter reuptake and increasing the dopamine and noradrenaline release by being taken up into neuronal cells and acting on the vesicular monoamine transporters [51, 61, 62]. Comparable cases using lisdexamfetamine have not been reported before.

### Reason 7: complex possible metabolic and genetic findings in psychiatric disorders

In the end the diagnosis of a CDG was not likely due to the finding of a transferrin mutation which was previously described in a healthy patient [63]. Polymorphisms in the transferrin gene have been found in patients with schizophrenia and were discussed to affect oligodendrocytes and myelin formation but the role in ADHD remains unclear [64]. Even if no clear evidence for a metabolic disorder was found it can be discussed as a reason for the development of psychiatric symptoms like in ADHD [65, 66]. Lastly, it is not clear what effect the found transferrin mutation might have concerning psychiatric symptoms and what other findings could have been made concerning metabolic diseases. The found mutation can be discussed as an incidental finding.

## Conclusion

This work refers to a case report but points towards problems of ADHD treatment in an outpatient clinic and overlaps with a possible SUD. A delayed start of medication was found in this case due to abnormal high biomarkers (CDT). Firstly, the patient rejected further diagnostic or treatment for the here found glycosylation abnormalities (possible CDG) and was satisfied with lisdexamfetamine due to an improvement with the therapy, but it needs to be discussed that in cases of metabolic disorders psychiatric symptoms can occur. Secondly, the later found transferrin mutation points towards a genetic case and the effects on psychiatric symptoms due to these findings are not clear. Further, we consider CDT as an important diagnostic tool in patients with ADHD because (beside relevance in alcohol consumption) overlaps with glycosylation abnormalities are possible. Lastly, the patient rejected to do a brain MRI scan, but the diagnosis of ADHD could be made based on extensive laboratory diagnostics and the lack of a clear recommendation for a brain MRI scan in the ADHD guidelines.

### Limitations

Due to a case report, there is a lack of generalizability. A confounding factor is the here described transferrin mutation which could be further discussed as a stigmatization for the patient regarding consumption of alcohol (high CDT). Missing further metabolic and genetic tests could have been useful to evaluate if the mentioned transferrin mutation is really affecting central nervous structures leading to symptoms of ADHD.

**Table 1:** Scores during diagnostic process

Test	Score	Cut-Off	Change in symptoms
ASRS-V1.1.	6 (2023)	minimum 4 symptoms with significant intensity	
ADHS-SB/HASE-SB	43 (2023) 34 (May 2024) 18 (August 2024)	18	Severe inattention and psychomotor agitation (2023). Psychomotor quieter but attention spans still reduced – disassembled a pen and smeared herself with ink during consultation (May 2024). Attention and psychomotor markable improved, thought form nearly appropriate (August 2024).
IDA-R	8 points childhood 7 severe symptoms for inattention 8 severe symptoms for hyperactivity (2023)	minimum 6 minimum 5 minimum 5	
WURS-K	35 (2023)	≥ 30	
BDI-II	15 (2023)	14–19 = mild Depression	
D2	Average concentration (2023)		
PSDI	slightly signs for personality accentuation: dependent, paranoid, optimistic, selfless (2023)		
AQ	29 (2023)	≥ 32	
MWT-B	IQ 94 (2023)		
GAF	40 (2023) 65 (2024)	Range 0-100% of function	Burdened by psychosocial stress related to work (2023). Improvement at work, finished exam at work (2024).

## Electronic supplementary material

Below is the link to the electronic supplementary material.

Supplementary Material 1

## Data Availability

Data is provided within the manuscript or supplementary information files.

## Abbreviations

ADHD: Attention deficit hyperactivity disorder
ADHS: Aufmerksamkeitsdefizit-/Hyperaktivitätsstörung
ADHS-SB: ADHS-Selbstbeurteilungsbogen
ALG8: Alpha-1,3-glucosyltransferase
AMA: Anti-mitochondrial antibodies
ANA: Anti-nuclear antibodies
AQ: Autism quotient
ASMA: Anti-smooth muscle antibodies
ASRS-V1.1: Adult ADHD self-reported scale
BDI-II: Becks depression inventory II
c-ANCA: Cytoplasmic anti-neutrophil cytoplasmic antibodies
CCDC115: Coiled-coil domain containing 115
CDG: Congenital disorder of glycosylation
CDT: Carbohydrate-deficient transferrin
CIWA-Ar: Clinical institute withdrawal assessment for alcohol-revised
COG: Conserved oligomeric Golgi
COPD: Chronic obstructive pulmonary disease
DAT: Dopamine transporter
DSM-IV: Diagnostic and Statistical Manual of Mental Disorders 4th edition
DSM-5: Diagnostic and Statistical Manual of Mental Disorders 5th edition
D2 test: D2 concentration-endurance test
EMA: European medicine agency
FDA: US food and drug administration
FUT8: Fucosyltransferase 8
HASE-SB: Homburger ADHS-Skalen für Erwachsene Selbstbeurteilungsbogen
HPLC: High-performance liquid chromatography
ICD-10-GM Version 2024: International statistical classification of diseases, 10th revision, German modification, version 2024
IDA-R: Integrated diagnosis of ADHD in adults revised version
LDH: Lactate dehydrogenase
LKM: liver kidney microsome antibodies
MPI: Mannose-6 phosphate isomerase
MRI: Magnetic resonance imaging
MWT-B: Multiple choice word test-B
p-ANCA: Perinuclear anti-neutrophil cytoplasmic antibodies
PGM1: Phosphoglucomutase-1
PGM3: Phosphoglucomutase-3
PSDI: Personality styles and disorder inventory
SLA: Anti-soluble liver antigen antibodies
SUD: Substance-use disorders
WHO: World health organization
WURS-K: Wender-Utah rating scale german short version
X-ANCA: Atypical anti-neutrophil cytoplasmic antibodies
